# Polypoid endometriosis of the Douglas pouch

**DOI:** 10.1016/j.radcr.2020.12.054

**Published:** 2020-12-30

**Authors:** A Jacquot, W Gertych, F Golfier, M Devouassoux-Shisheboran, P Rousset

**Affiliations:** a Radiology Department, Lyon Sud University Hospital, Hospices Civils de Lyon, Lyon, France; b Gynaecology and Obstetrics Department, Lyon Sud University Hospital, Hospices Civils de Lyon, Lyon, France; c Lyon 1 Claude Bernard University, Lyon, France; d Pathology Department, Lyon Sud University Hospital, Hospices Civils de Lyon, Lyon, France

**Keywords:** Endometriosis, Neoplasm, Peritoneal diseases, Magnetic resonance imaging

## Abstract

Polypoid endometriosis is a rare form of endometriosis that corresponds to a benign variant but which systematically mimics malignant tumors. Magnetic resonance imaging (MRI) is the preferred imaging modality for these lesions. We present herein a case of a 43-year-old female with recent pelvic pain and longstanding dyspareunia related to polypoid endometriosis of the Douglas pouch. MRI found an infiltrative lesion 6 cm in diameter with intermediate signal on T2-weighted imaging, cystic hemorrhagic spots, and fibrous surrounding rim of nodular portion. There was no functional sign of malignancy (no diffusion restriction, pronounced tumor enhancement, or metastasis). The patient underwent total abdominal radical colpohysterectomy with bilateral salpingectomy and ovarian transposition was performed. Histopathological examination found a multinodular endometrial-type polypoid mass arising from the serosa of the cervix, with cystic area and fibrous surrounding tissue. In the case presented, MRI findings were useful for preoperative diagnosis that altered patient management by supporting a complete but reasonable surgical resection that yielded relief of symptoms.

## Introduction

Polypoid endometriosis is a rare form of a very common disease affecting women of reproductive age that mimics a neoplasm on clinical, surgical, and gross examination [1]. Preoperative imaging diagnosis is also challenging. However, magnetic resonance imaging (MRI) may play a pivotal role for a timely and accurate diagnosis. We present herein a case of a 43-year-old female with key MRI features of a polypoid endometriosis of the Douglas pouch and correlate each MRI sign to the surgical and pathological findings.

## Case-report

**Fig. 1:**
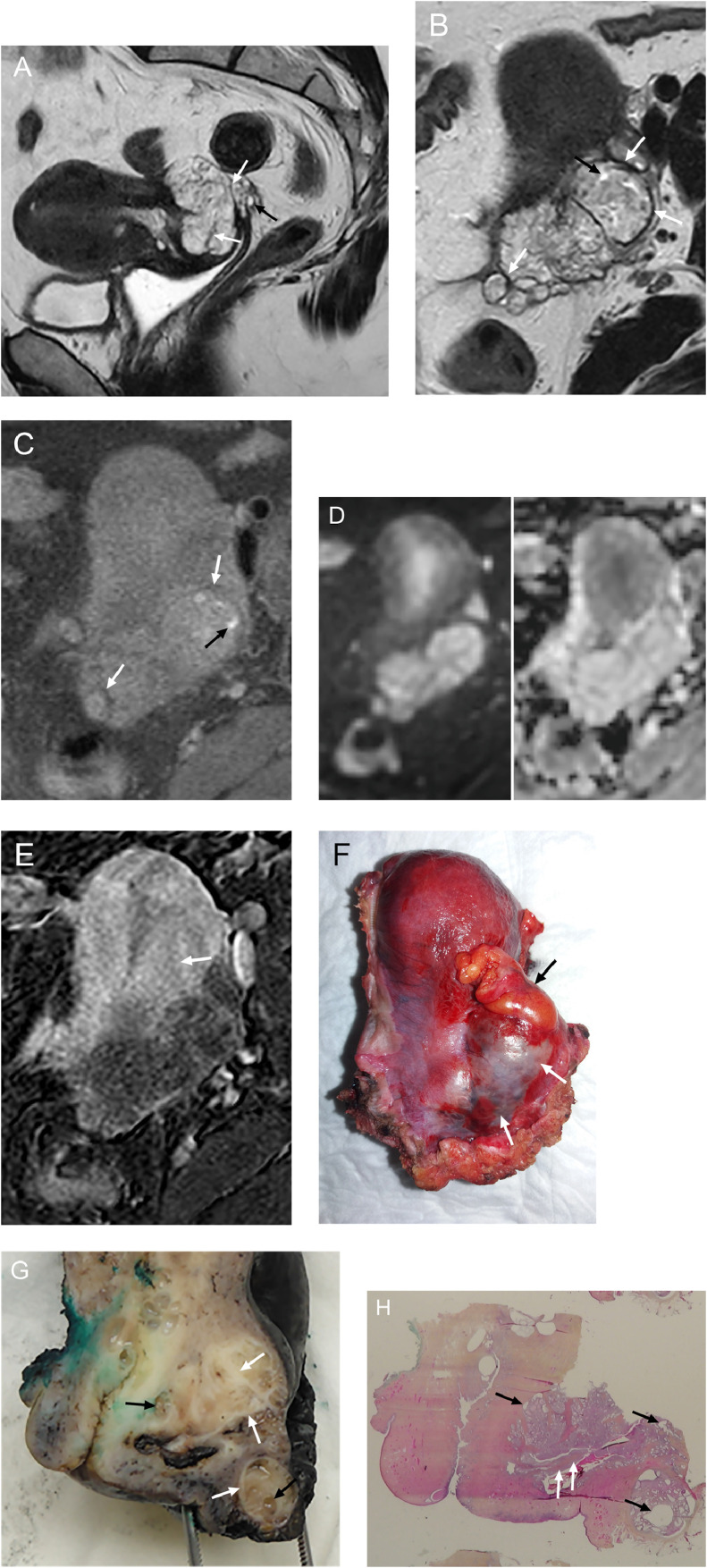
A 43-year-old nulliparous woman with polypoid endometriosis of the Douglas pouch. A. Sagittal T2, B. axial T2 and C. axial T1 with fat saturation-weighted images show a heterogeneous T2 intermediate signal mass with cystic areas, some being hemorrhagic (black arrows). This reflects the presence of abundant dilated endometrial-like glands detected on histology (G). T2 and T1 hypointense septations and a peripheral hypointense rim-like alterations (white arrows) are present. D. Axial diffusion (b = 1000 s/mm²; left) and apparent diffusion coefficient (ADC) map (generated from b value of 0 and 1000 s/mm²; right) images show no restricted diffusion and high mean ADC value (measured in the largest nodular portion: 2.15 × 10^−3^ mm²/s). E. Axial subtraction of late-phase postcontrast T1-WI shows weak enhancement of the mass compared with that of uterine myometrium (arrow) and mild enhancement of septations and peripheral rim. F. Sagittal view (photograph) of the hysterectomy piece shows a reddish mass (white arrows) infiltrating the pouch of Douglas and the posterior cervix. Note the adhesive epiploic appendage of the rectosigmoid (black arrow). G. Macroscopy (photograph) shows a multinodular whitish-yellow polypoid mass arising from the serosa of the cervix (green coloration indicating the anterior part of the cervix), with cystic area (black arrows) and fibrous surrounding tissue (white arrows) corresponding to fibrous structures found on MRI (panels A, B, C, and E). H. Histopathological photomicrograph at 1.5x magnification (hematoxylin-eosin-saffron stain) shows numerous endometrial glands with cystic dilatation (black arrows) and some focal polypoid protrusions at the serosal surface (white arrows).

A 43-year-old nulliparous woman consulted for mild metrorrhagia that had started 3 months earlier, she also suffered from recent pelvic pain and longstanding dyspareunia. Her medical history was unremarkable. Physical examination found a complete obliteration of the Douglas pouch with a hard and enlarged supravaginal cervix. MRI (1.5T) showed a lobulated nodular mass up to 6 cm in diameter, arising from the Douglas pouch with a large extrinsic infiltration of the posterior wall of the cervix, the posterior vaginal fornix, as well as perirectal and pericervical fat (Fig. 1). The mass was heterogeneous with mostly intermediate signal on T2-Weighted Images (WI) and hyperintense cystic areas, few of which being hyperintense on T1-WI consistent with hemorrhagic content. Some hypointense septations and a peripheral hypointense rim-like alteration on T2-WI were present. Apparent Diffusion Coefficient map showed no restricted diffusion. The mass was hypovascular during each phase of dynamic contrast-enhanced T1-WI (type 1 curve compared with that of uterine myometrium) and with a late enhancement of septations and peripheral rim. MRI showed no other pelvic abnormalities. As a standard cervical biopsy was uninformative, a trucut biopsy was performed under general anesthesia and analysis evoked usual endometriotic lesion and there was no evidence of malignancy; this remained consistent with MRI findings suggesting polypoid endometriosis. After multidisciplinary team meeting, the patient underwent total abdominal radical colpohysterectomy with bilateral salpingectomy to relieve the pelvic pain. The mass, fixed to the posterior wall of the cervix and infiltrating perirectal fat, was completely removed en bloc resection with the uterus. Additionally, given the patient's young age, an ovarian transposition was performed. Gross examination found a multinodular and polypoid appearance, with microcystic reshaping and a slightly mucoid content, well limited and reaching the adipose tissue focally. Histopathological examination found numerous and hyperplastic benign endometrial-type glands showing glandular crowding and cystic dilatation, without nuclear atypia. The endometrial stroma was cellular without nuclear atypia but demonstrating periglandula cuffs of increased stromal cellularity. Focal intraglandular polypoid stromal protrusions were seen, and polyps with fibro-vascular cores and simple hyperplasia of the glandular epithelium were visible at the peritoneal surface. These histopathological findings were consistent with polypoid endometriosis; the absence of prominent phyllodes architecture, nuclear atypia of the stromal component, and heterologous mesenchymal elements helped rule out an adenosarcoma. At 24 months of follow-up symptoms were completely relieved.

## Discussion

Polypoid endometriosis is a benign variant of endometriosis that systematically mimics malignant tumors due to its infiltrative pattern. The ovaries, colon, and uterine serosa are among the most common locations affected by polypoid endometriosis [1]. While classic endometriosis predominates in premenopausal women, polypoid endometriosis more commonly affects peri- to postmenopausal women. Preoperative diagnosis is difficult, particularly in the absence of previous history of endometriosis or other endometriotic MRI sign, as in the case presented herein. However, based on our detailed description of the case presented, with surgical and pathological correlations, together with the few recent imaging reports [2], [3], [4], [5], several features can aid to narrow the diagnosis irrespective of the location. Positive suggestive morphological signs are intermediate signal of the mass on T2-WI with the presence of cystic hemorrhagic spots on T1-WI and a fibrous surrounding rim of the lesion. The negative functional signs of malignancy are the lack of diffusion restriction and pronounced dynamic enhancement. These latter signs, together with the absence of ancillary features such as lymphadenopathy or peritoneal metastases, may help to rule out a malignant transformation of an endometriotic lesion or other malignant differential diagnosis. Presence of imaging stigmata of endometriosis, not present in the case presented, may also reinforce the diagnosis, but the definitive diagnosis relies on histopathological examination. MRI, by suggesting the diagnosis, can alter the decisions taken for patient management during a multidisciplinary team meeting by either supporting hormonal suppressive treatment after confirmation by biopsy, or defining the anatomical relationship for a complete but reasonable surgical resection that yields relief of symptoms and good prognosis.

## Consent

Informed written consent for publication was obtained from the patient. The case report was in accordance with the 1964 Helsinki declaration and later amendments, and informed patient was obtained. All the authors significantly contributed to this work and agree with its content.
